# Integrating Somatic Mutations for Breast Cancer Survival Prediction Using Machine Learning Methods

**DOI:** 10.3389/fgene.2020.632901

**Published:** 2021-01-18

**Authors:** Zongzhen He, Junying Zhang, Xiguo Yuan, Yuanyuan Zhang

**Affiliations:** ^1^School of Computer Science and Technology, Xidian University, Xi’an, China; ^2^School of Information and Control Engineering, Qingdao University of Technology, Qingdao, China

**Keywords:** breast cancer, multi-omics, survival prediction, somatic mutation, mRMR, MKL

## Abstract

Breast cancer is the most common malignancy in women, and because it has a high mortality rate, it is urgent to develop computational methods to increase the accuracy of breast cancer survival predictive models. Although multi-omics data such as gene expression have been extensively used in recent studies, the accurate prognosis of breast cancer remains a challenge. Somatic mutations are another important and promising data source for studying cancer development, and its effect on the prognosis of breast cancer remains to be further explored. Meanwhile, these omics datasets are high-dimensional and redundant. Therefore, we adopted multiple kernel learning (MKL) to efficiently integrate somatic mutation to currently molecular data including gene expression, copy number variation (CNV), methylation, and protein expression data for the prediction of breast cancer survival. Before integration, the maximum relevance minimum redundancy (mRMR) feature selection method was utilized to select features that present high relevance to survival and low redundancy among themselves for each type of data. The experimental results demonstrated that the proposed method achieved the most optimal performance and there was a remarkable improvement in the prediction performance when somatic mutations were included, indicating that somatic mutations are critical for improving breast cancer survival predictions. Moreover, mRMR was superior to other feature selection methods used in previous studies. Furthermore, MKL outperformed the other traditional classifiers in multi-omics data integration. Our analysis indicated that through employing promising omics data such as somatic mutations and harnessing the power of proper feature selection methods and effective integration frameworks, the breast cancer survival predictive accuracy can be further increased, thereby providing a more optimal clinical diagnosis and more effective treatment for breast cancer patients.

## Introduction

Breast cancer is the most common malignant tumor in women. Although there are millions of breast cancer survivors in the United States, breast cancer is the main cause of cancer-related deaths worldwide because of its high mortality rate (Ferlay et al., 2010). Thus, it is urgent to design highly accurate methods to predict the survival of breast cancer patients. Accordingly, effective survival predictors could finally contribute to the reduction of the overall mortality of breast cancer and could further improve the life quality and increase the lifespan of breast cancer patients.

Recently, the Cox regression model (Yuan et al., 2014; Xu et al., 2016) and traditional machine learning classification methods, such as support vector machine (SVM) (Xu et al., 2013), Bayes classifier (Gevaert et al., 2006), and random forest (RF) (Nguyen et al., 2013), have been widely deployed to identify breast cancer prognostic biomarkers. Multiple survival prediction models have been mainly developed based on gene expression data. The Cancer Genome Atlas (TCGA) (Cancer Genome Atlas Research Network, 2013; Brennan et al., 2014) provides multiple types of molecular data such as gene expression (Exp), copy number variation (CNV), methylation (Methy), protein expression (Protein), and somatic mutation (SM) data for various cancers, including breast cancer. Moreover, the advancement of machine learning technologies enables various data types to be combined within a model (Chen et al., 2019; Lan et al., 2020), which may increase the accuracy of predictive models.

One of the biggest challenges in breast cancer research involves the effective combination of heterogeneous data sources into survival prediction models, making the selection of a proper integration method essential. In previous studies (Seoane et al., 2014; Zhang et al., 2016; Sun et al., 2018; Zhang A. et al., 2019; Zhang Y. et al., 2019), multiple kernel learning (MKL) (Lanckriet et al., 2004; Rakotomamonjy et al., 2008; Kloft et al., 2011) was successfully used to integrate different types of data into a universal model to distinguish short-term and long-term cancers survivors. MKL uses different kernels for different types of data, and then trains the weight of each kernel to select the best combination of kernel functions for classification. These studies have demonstrated that models that were obtained using integrated data improved the performance of survival prediction compared to models that used only one single data type.

A previous study (Sun et al., 2018) showed that MKL outperformed Cox-based regression models for breast cancer survival prediction. However, omics data, such as Exp, CNV, and methylation data, are usually extremely high-dimensional and redundant (Dey et al., 1990). In the previous study (Sun et al., 2018), information gain ratio (IGR) was utilized to select survival relevant features from multi-omics data, but the redundancy of dataset features was not considered. Despite the promising performance of the above MKL-based studies for breast cancer prognosis, somatic mutations are rarely considered for breast cancer survival prediction due to their complexity and heterogeneity in serious disease. Therefore, there is still much room to increase the accuracy of breast cancer survival models by incorporating somatic mutations into the MKL model.

Currently, somatic mutations are strongly correlated with the clinical symptoms of breast cancer (Griffith et al., 2018), and they have been successfully adopted for the classification of primary cancer sites (Chen et al., 2015) and identification of survival-related cancer subtypes (Hofree et al., 2013; He et al., 2017; Ronen et al., 2018; Arslanturk et al., 2020). Somatic mutations are sparse but common mutations of that offer less accuracy in the prediction of cancer survival (Zhang et al., 2018; Ye et al., 2019). Previous studies (Haricharan et al., 2014; Griffith et al., 2018; Zhang et al., 2018; Ye et al., 2019) have reported that mutations enriched in specific pathways have shown potential for breast cancer survival prediction. The authors of a previous study (Griffith et al., 2018) stated that uncommon recurrent somatic mutations should be further explored to explain breast cancer survival outcomes. In the present study, the effect of somatic mutations on the integrated prognosis of breast cancer is explored.

In the present study, we applied the state-of-the-art MKL method in the integration of somatic mutation datasets with previously used omics data, including Exp, CNV, Methy, and Protein, to train and test an integrated breast cancer survival prediction model. The maximum relevance minimum redundancy (mRMR) algorithm (Ding and Peng, 2005; Radovic et al., 2017) was used to alleviate the redundancy of the data, by simultaneously selecting highly predictive but non-redundant features from each type of molecular data. Then, selected features from multiple data type were integrated into the MKL classification.

In order to gauge the performance of our method, first, the newly introduced method was compared with different single data types and integrated datasets to verify the effectiveness of somatic mutations, and the results indicated that there was a remarkable improvement in the prediction performance when somatic mutations were included. Different feature selection algorithms were then studied, and the experimental results demonstrated that mRMR was the most optional among them. Furthermore, the MKL classification method was compared with other traditional classifiers, and the experimental results proved the superiority of MKL in data integration. Finally, the newly introduced model was validated in an independent validation dataset and achieved a promising high accuracy in survival prediction. According to the results, the most optimal performance was achieved by our method, which demonstrated the feasibility of integrating somatic mutations in the prognostic models and the usefulness of mRMR and MKL in breast cancer prognosis.

The reminder of this article is organized as follows. A workflow of our proposed method and related methods are described. Next, comparative studies were carried out to evaluate the performance of the proposed methods and their comparison methods, as well as to analyze the most informative features discovered by our model. Then, we applied our model on the validation dataset. Finally, the proposed method is discussed, and it is expected to undergo the improvement in future studies.

## Materials and Methods

### Workflow of the Proposed Method

**FIGURE 1:**
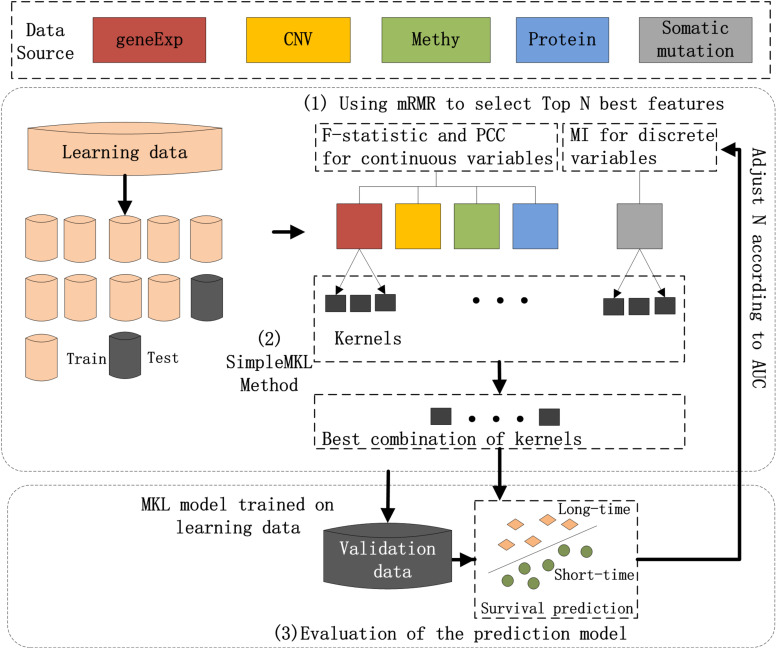
Workflow of the hybrid combination of the MKL model with the mRMR feature selection method to integrate five types of molecular data for the prognosis of breast cancer. (1) The most N-informative features were separately selected using the mRMR method for each type of data in the learning dataset; (2) SimpleMKL with 10-fold cross-validation was deployed on the learning dataset for breast cancer prognosis to train an optimal model; and (3) the prediction model on learning dataset and the validation dataset were evaluated.

The workflow chart of the proposed method is shown in Figure 1. Preprocessing of the input dataset initially occurred, during which entire datasets were randomly divided into a learning dataset (80% of the entire dataset) and validation dataset (20%). Then, three main steps were carried out to realize the prediction of breast cancer prognosis.

The three main steps include: (1) The most N-informative features were separately selected using the mRMR method for each type of data in the learning dataset; (2) SimpleMKL with 10-fold cross-validation was deployed on the learning dataset for breast cancer prognosis to train an optimal model; and (3) the prediction model on learning dataset and the validation dataset were evaluated for their ability to learn data. A detailed description of each of the steps is listed below.

### Data Input and Preprocessing

The Cancer Genome Atlas provides multiple types of biomolecular data. High-level molecular data for breast cancer were retrieved from TCGA, including gene expression, gene CNV, gene methylation, protein expression, and somatic mutation along with clinical features from the University of California Santa Cruz (UCSC) cancer browser website^1^ (Mary et al., 2014). The downloaded dataset consisted of five types of data, including different numbers of samples, and the original data matrixes were structured with rows denoting patient samples and columns denoting features. A total of 139 true normal, seven metastatic, and 13 male patients’ samples were removed, and regarding somatic mutations, samples with less than 10 mutations were removed (Hofree et al., 2013; He et al., 2017). We finally obtained 488 primary breast tumors together with survival time, and all samples of them included all of the five aforementioned genomic data types. The details of our dataset are illustrated in Table 1. The median age at diagnosis was 57.37, and the median survival time was 42.43 months, which is in agreement with the previous research (Sun et al., 2018).

**TABLE 1:** The detailed information in our breast cancer dataset.

Properties	Number
Total population of primary cancer	488
Long-term survivors	119
Short-term survivors	369
Mean age at diagnosis (years old)	57.37
Median survival (months)	42.43

We followed the protocol from our previously published studies (He et al., 2017, 2019), and we first removed the genes with missing values in more than 10% of samples for gene expression, CNV, gene methylation, protein expression, and somatic mutations. After that, flat variables that had the same values in more than 80% of the samples (non-informative) were discarded except in the case of somatic mutations (Yuan et al., 2014; He et al., 2019). According to the previous study (He et al., 2019), the RNA-Seq gene expression level 3 transcription was log2 transformed and RSEM-normalized (Li and Dewey, 2011). Regarding the CNV features, we directly utilized the gene-level copy number values that were estimated using the GISTIC2 method (Mermel et al., 2011; Yuan et al., 2017; Yuan et al., 2019, 2020a,b). For gene methylation and protein expression, we directly used the original data with z-score normalization. For somatic mutation, we also directly utilized the original binary data, and in addition, genes that were mutated in more than one sample were reserved for further analysis. The gene expression, CNV, gene methylation, and somatic mutations contained 18,000, 25,000, 22,000, and 14,000 features, respectively, after data filtering, and the properties of these datasets are shown in Table 2.

**TABLE 2:** The properties of five types of genomic data for our breast cancer prediction.

Data types	Feature number
Gene expression	18624
CNV	24774
Gene methylation	21136
Protein expression	170
Somatic mutations	13602

In the present study, the survival prediction for breast cancer was defined as a binary classification problem with a threshold of 5 years as conducted in previous studies (Seoane et al., 2014; Zhang et al., 2016; Sun et al., 2018; Zhang A. et al., 2019). Of the total, 369 out of the final 488 patients with survival shorter than 5 years were considered as short-term survivors, and 119 patients with survival longer than 5 years were considered as long-term survivors. Moreover, the long-term patients were labeled as 1, while short-term patients were labeled as 0. After the initial data preprocessing, the entire dataset was randomly divided into the learning dataset (80%) and validation dataset (20%). For each type of data, we initially conducted the following feature selection on the learning dataset containing 390 breast cancer patients, and trained and tested the integrated MKL model on it to obtain the optimal parameters. Then, we applied the optimal model on the validation dataset that included 98 patients.

### mRMR Feature Selection

Five different types of genomic data were used in the present study, as described above, and the number of variables for most types of genomic data exceeded 10,000 after feature preprocessing. However, this large number of features may cause poor performance due to dimensionality and high redundancy (Jain and Zongker, 1997; Jie et al., 2015). Therefore, according to our previous study (He et al., 2019), mRMR was adopted in the present study to select the most useful features for the prognostic model.

The mRMR is a feature selection method that aims to select a subset of features that are highly related to the output classes and have low redundancy between them (Radovic et al., 2017). In the present work, mRMR was deployed to select features from five types of molecular data that are the most highly relevant with respect to survival and the least correlated among themselves. Then, the most relevant features for each molecular dataset were combined to form a candidate feature set to be used for classification. A feature of one type of genomic dataset for the *i*th variable with N individuals is denoted as *v*_*i*_ ∈ *R^M^*,*i* = 1,…,*M*, and the survival prediction labels with N individuals as *l* ∈ *R*. For label *l*, mRMR aims to search a feature subset *S* with *k* features{*v*_*i*_}, which collectively have the maximal relevance (Max-Relevance) Rel(*S*,*l*)on the target label *l* and the minimal redundancy (Min-Redundancy) Red(*S*).

The F-statistic (F) was used to calculate the relevance between feature variables with binary survival terms and the Pearson correlation coefficient (PCC) was used to measure the redundancy for the continuous feature variables of the gene expression, CNV, gene methylation, and protein datasets. Max-Relevance is defined in Eq. 1, where relevance Rel(*S*,*l*) is calculated using the mean value of all F-statistic values *F* of the individual variables *v*_*i*_ with the label *l*. In parallel, the Min-RedundancyRed(*S*) constraint was adopted to select irrelevant features, and is shown as Eq. 2.

(1) $$\max\text{Rel}\,\left(S,l\right),\mathrm{Rel}=\frac{1}{\left|S\right|}\,\sum_{v_{i}\in S}F\,\left(v_{i};l\right),$$

(2) $$\min\text{Red}\,\left(S\right),\mathrm{Red}=\frac{1}{{\left|S\right|}^{2}}\,\sum_{v_{i},v_{j}\in S}P\,C\,C\,\left(v_{i};v_{j}\right)$$

For binary discrete feature variables of somatic mutation data, the mutual information (MI) was used to calculate both the relevance between feature variables and survival terms, and the redundancy between mutations. Max-Relevance is used to select features satisfying Eq. 3, where relevance Rel(*S*,*l*) is obtained by the mean value of all MI values of individual variable *v*_*i*_ with label *l*. The Min-Redundancy constraint Red(*S*) is used to select irrelevant features, and is shown as Eq. 4.

(3) $$\max\text{Rel}\,\left(S,l\right),\mathrm{Rel}=\frac{1}{\left|S\right|}\,\sum_{v_{i}\in S}M\,I\,\left(v_{i};l\right),$$

(4) $$\min\text{Red}\,\left(S\right),\mathrm{Red}=\frac{1}{{\left|S\right|}^{2}}\,\sum_{v_{i},v_{j}\in S}M\,I\,\left(v_{i};v_{j}\right)$$

Finally, as shown in Eq. 5, the operator ϕ(Rel,Red) was deployed to simultaneously optimize the two constraints “Max-Relevance” and “Min-Redundancy” based on the MI quotient (MIQ) criterion (Radovic et al., 2017; He et al., 2019) to obtain the best feature subsets, as shown in Eq. 5:

(5) $$\max_{v_{k}}\phi \,\left(\text{Rel},\text{Red}\right),\,\phi =\text{Rel}/\text{Red}$$

The area under the curve (AUC) value is used as a metric to evaluate the performance and the most optimal number of the most relevant and non-redundant features *k* for each data type was determined by comparing the AUC valued for the models. After the mRMR features were selected for each type of genomic data, the most informative features were combined and used as the input feature set for the classification problems.

### Multiple Kernel Learning

In our study, we aimed to integrate multiple types of genomics data, with a focus on somatic mutations. Although the fusion of multiple types of data into one model is one of the most widely used methods for classification, this is not feasible due to the fact that different types of molecular data present different feature representations (Khademi and Nedialkov, 2016). MKL has become a natural method to enhance the interpretability of models and to address the data integration problem. The optimal function can be obtained by constructing a linear weighted combination of predefined *M* kernels. The optimal combination of kernels is given as Eqs 6 and 7:

(6) $$K\,\left(x_{i},x_{j}\right)=\sum_{\text{m}=1}^{M}d_{m}\,K_{m}\,\left(x_{i},x_{j}\right),$$

(7) $$s.t.\,d_{m}\geq 0,\mathrm{and}\,\sum_{m=1}^{M}d_{m}=1,$$

where *d*_m_ denotes the weight of the *m*th different kernel *K*_*m*_(*x*_*i*_,*x*_*j*_).

Some methods based on MKL have been proposed and many of them outperformed uni-MKL (Rakotomamonjy et al., 2008; Gönen and Alpaydin, 2011; Kloft et al., 2011). However, most of the weights *d*_*m*_ of the kernels were 0 and thus non-contributory to the MKL model (Ikonomov et al., 2013). In the present work, SimpleMKL (Zhang et al., 2016), which is based on a weighted L2-norm regularization and is more powerful than other methods (Yan et al., 2009), was adopted as our classification model. It employs dual kernels in the of classic kernel optimization problem, which can be presented as Eq. 8:

(8) $$f\,\left(x\right)=\sum_{i=1}^{l}\alpha_{i}^{*}\,K\,\left(x_{j},x_{i}\right)+b^{*}$$

The decision function is given as:

(9) $$\begin{array}{l} \underset{f,b,\varepsilon }{\text{min}}\frac{1}{2}f_{H}^{2}+C\sum_{i}\varepsilon_{i}\\ s.t.\;y_{i}(f(x_{i})+b)\geq 1-\varepsilon_{i}\forall_{i},\\ \varepsilon_{i}\geq 0\;\;\;\;\;\;\;\;\;\;\;\forall_{i} \end{array}$$

where || f||_*H*_ denotes a kernel in Hilbert space related to a kernel *K*_*m*_. The overall kernel can be divided into different kernels, and we replace || f||_H_ with ∑_*m*_|| f_*m*_ ||_*HM*_ to obtain:

(10) $$\begin{array}{l} \underset{f_{m,b,\text{ε,d}}}{\text{min}}\frac{1}{2}\underset{m}{\Sigma }||f_{m}|{|}_{HM}^{2}+C\sum_{i}\varepsilon_{i}\\ s.t.\;y_{i}\underset{m}{\Sigma }f_{m}(x_{i})+y_{i}b\geq 1-\varepsilon_{i}\forall_{i},\\ \varepsilon_{i}\geq 0\;\;\;\;\;\;\;\;\;\;\;\;\;\forall_{i}\\ \underset{m}{\Sigma }d_{m}=1,d_{m}\geq 0\;\;\;\;\;\;\;\;\forall_{m} \end{array}$$

Optimization matter is performed using the convex optimization mathematical algorithm (Rakotomamonjy et al., 2008). Using multiple kernels increases the decision the power of the decision function and also increases the prediction performance compared to using one single kernel. In the present study, SimpleMKL was deployed to integrate five different types of molecular data including gene expression, CNV, gene methylation, protein expression, and somatic mutation.

Considering the number of data types used in our study, five different kernels were independently built and further integrated into a generic model. Each kernel corresponds to each individual data type (gene expression, CNV, gene methylation, protein expression, and somatic mutation). The “Poly” (Eq. 11) polynomial base kernel with a search range of degrees of freedom *d*{1 2 3} (Seoane et al., 2014) and the “Gaussian” (Eq. 12) kernel with a search range of the parameter δ {0.25 0.5 1 2 5 7 10 12 15 17 20} (Zhang et al., 2016; Sun et al., 2018) were used as kernel types.

(11) $$K\,\left(x_{i},x_{j}\right)={\left(x_{i}^{T}\,x_{j}+1\right)}^{d},$$

(12) $$K\,\left(x_{i},x_{j}\right)=\exp\left(-\frac{{||x_{i}-x_{j}||}^{2}}{2\,\delta^{2}}\right)$$

In summary, the SimpleMKL directly addressed a multiple kernel SVM optimization problem and greatly reduced computation costs when compared to the use of learning kernel combinations from individual kernels.

### Evaluation

The dataset used in our study was randomly divided into learning and validating sets in order to assess the performance of the proposed method. For the learning set, we used mRMR to select the most optimal features and to determine the model through 10-fold cross-validation experiments. Then, the pre-trained MKL model and its optimal parameters were used to predict the validation set. Because the validation dataset was not used in the cross-validation process, the model derived from the learning dataset was tested on an independent validation dataset.

To assess the performance of our model, AUC, the most widespread evaluation metric for classification problems, was used to assess the performance of the proposed model. AUC is defined as the area under the receiver operating characteristic (ROC) curve, and it is used to quantify the overall performance of a classification model. Specifically, AUC = 1 denotes perfect performance, and 0.5 denotes random guessing. Pre (precision, Eq. 13), Sn (sensitivity, Eq. 14), Sp (Specificity, Eq. 15), and Acc (Accuracy, Eq. 16) were also employed in addition to AUC as classification performance metrics for breast cancer prognosis. The definitions of those metrics are provided below:

(13) $$P\,r\,e=\frac{T\,P}{T\,P+F\,P},$$

(14) $$S\,n=\frac{T\,P}{T\,P+F\,N},$$

(15) $$S\,p=\frac{T\,N}{T\,N+F\,P},$$

(16) $$A\,c\,c=\frac{T\,P+T\,N}{T\,P+T\,N+F\,N+F\,P}$$

where TP, FP, TN, and FN denote true positive, false positive, true negative, and false negative, respectively.

## Results

### Comparison Studies on Learning Datasets

The proposed method was compared with other methods in three different applications: (1) comparison of the results of the models with different datasets based on the same method; (2) comparison of the results of different feature selection methods under the same datasets; and (3) comparison of the integration results of classification methods, under the same integrated datasets. AUC was used as an evaluation metric when comparing different methods and 10-fold cross-validation was applied for all methods.

#### Comparison of ECMPS and Other Data Types

Seven different MKL-based models were built using five single types of molecular data [gene expression (Exp), CNV, gene methylation (Methy), protein expression (Protein), and somatic mutations (SM)] and two integrated datasets with and without somatic mutation data in order to evaluate the role of somatic mutations in breast cancer survival prediction. The dataset integrating gene expression, CNV, gene methylation, and protein expression is abbreviated as “ECMP,” and the dataset integrating all five molecular datasets including somatic mutations is denoted as “ECMPS.”

**FIGURE 2:**
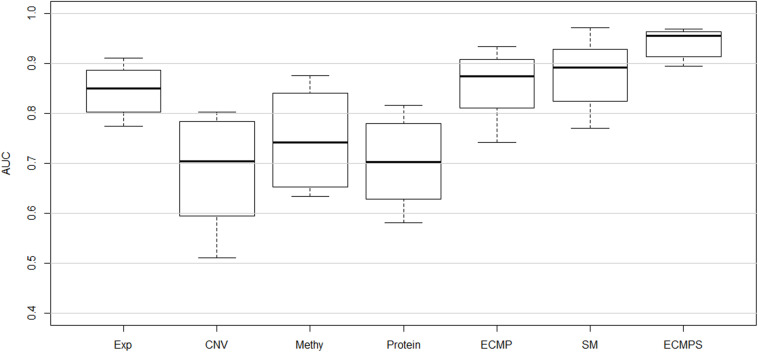
Performance of classifying long-term and short-term survivors from a breast cancer dataset using different types of data based on the proposed hybrid combination of mRMR feature selection and MKL classification methods.

**FIGURE 3:**
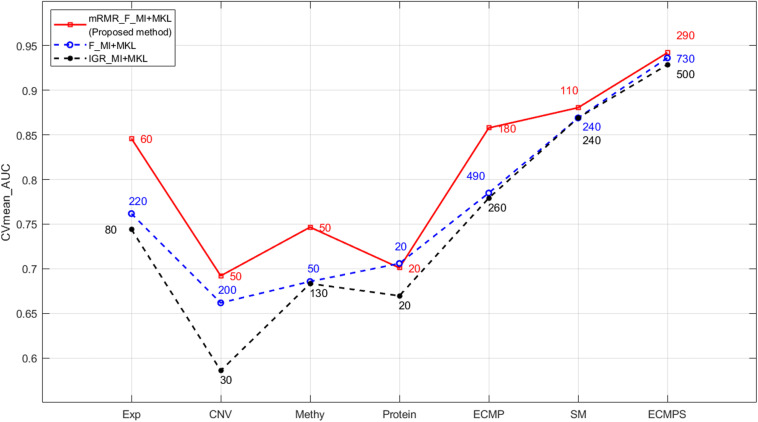
Performance comparison of mRMR and the two k-best methods based on MKL under different data types. The numbers in different colors on the lines indicate the number of optimal features selected by the corresponding method.

The corresponding mean of the AUC value of 10-fold cross-validation (CVmean_AUC) for each of the seven models, using the mRMR feature selection and the MKL classification method, was calculated to compare the predictive performance of breast cancer survival models. The results are displayed in Figure 2, with the mean values of the boxplots corresponding to the red line in Figure 3. As shown in Figure 3, the ECMPS model consistently exhibited significantly more optimal performances than all the other models for all three feature selection methods. The two integrated models present obvious improvements compared to the single data type model results, suggesting that integrated models are more optimal than single data type ones, which is consistent with previous studies (Zhang et al., 2016; Sun et al., 2018).

In Figure 2, the mean value of the AUC for the multi-data ECMP model without somatic data is 0.8854, and the corresponding value for the ECMPS model increased to 0.9421 when incorporating somatic mutation. In addition, among the single data type models, the AUC of the somatic mutation model was higher than that of the model using the other four single data types and ECMP. Thus, our experimental results indicated that the somatic mutation data is able to increase the accuracy of the survival prediction for breast cancer patients.

**FIGURE 4:**
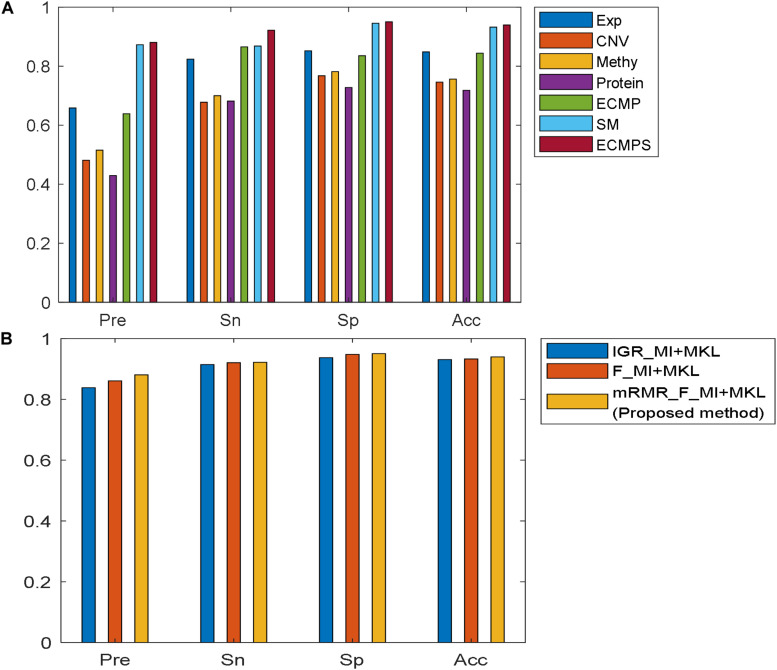
Comparison of performances of the models using different evaluation metrics: Pre, Sn, Sp, and Acc. **(A)** Performance of the proposed method in seven datasets. **(B)** Performance of various feature selection methods based on MKL under the same data type “ECMPS.”

The Pre, Sn, Sp, and Acc values for each dataset model were calculated in addition to the AUC based on the proposed method, and the results are presented in Figure 4A. Figure 4A shows that the integrative models combining different types of data, including somatic mutations, overcome the models using single data types for classification. The experimental results indicated that the proposed integrated model can successfully predict the survival time for breast cancer patients and somatic mutations can improve predictive accuracy.

#### Comparison of mRMR With Different Feature Selection Methods

We used mRMR to select the variables for each of the five types of molecular data. Then, the features with the largest relevance to the survival and lowest redundancy among themselves were selected, and they were combined as integrated features using the MKL classification model. The most optimal number of selected non-redundant features *k* for each molecular data type was determined by comparing the AUC values in the prediction results. According to the number of features reported in the previous study (Sun et al., 2018), we set *k* = [10, 20,…,300] in our work and chose the optimal parameter *k* as the final parameter for each data type in our study based on the prediction result.

**FIGURE 5:**
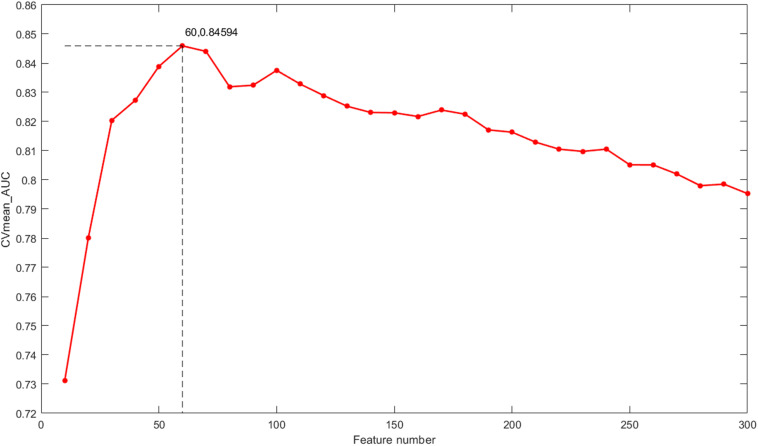
The mean value of the AUC for 10-fold cross-validation (CVmean_AUC) under the feature numbers ranging from 10 to 300 for the model based on gene expression.

The classification outcomes of the five data types under different parameters are presented in Supplementary File S1. The optimal feature number was selected based on the position of the maximum AUC value as the final parameter for a model of further integration. Take gene expression for example, as shown in Figure 5, the optimal number of features in the gene expression model using the proposed method is 60, which achieves the largest mean value of AUC with 10-fold cross-validation. Finally, we chose *k* = [60, 50, 50, 20, 110] as the optimal parameters for the five types of molecular data (Exp, CNV, Methy, Protein, and SM), respectively, for further integration analysis, and the total 290 features were obtained for our integrated ECMPS model.

The F-statistic (F) and PCC were used for the mRMR feature selection method to calculate the relevance and redundancy (Radovic et al., 2017), respectively, for four continuous data types, including Exp, CNV, Methy, and Protein, in order to maintain the original information for different types of data. MI was used to calculate both the relevance and redundancy of somatic mutation features, and is short for “mRMR_F_MI.” In all cases, the selected features were integrated using MKL classification. To assess the performance of the mRMR feature selection method in the selection of features for our breast cancer survival prediction model, the proposed mRMR feature selection was compared with two commonly used k-best methods, which only consider relevance with the output, based on the same datasets and classification method MKL: (1) F-MI. Compared to the proposed method, it only uses the F-statistic and MI to select the most optimal k-best features for four continuous molecular datasets and discrete somatic mutation. (2) IGR-MI. It adopts a recently used feature selection method, the IGR (Sun et al., 2018), for four continuous molecular datasets and MI for discrete somatic mutation.

The proposed mRMR method outperformed both k-best feature selection methods F-statistics and IGR for four continuous molecular data types and their integration ECMP model according to the results shown in Figure 3. For instance, 260 features were selected by IGR based on the ECMP model and the AUC value was 0.7791, which was consistent with previous studies (Sun et al., 2018). Next, 180 features were selected using mRMR and AUC was 0.8578 showing that mRMR can achieve higher predictive accuracy using fewer features. The mRMR method also outperformed MI for discrete somatic mutation returning a smaller number of features. The most optimal result was obtained by mRMR and the total integration model ECMPS. The metrics Pre, Sn, Sp, and Acc were calculated in addition to the AUC for each dataset model, with a more optimal performance by mRMR as compared to the other the two k-best methods (Figure 4B). Our findings indicated that the use of proper feature selection methods is crucial to the classification process.

As the red line shows in Figure 3, for the integrated ECMPS model, 290 features were selected as more relevant to survival and non-redundant features in the integrated ECMPS mode consisting of 60 Exp, 50 CNV, 50 Methy, 20 Protein, and 110 SM using mRMR, and the most optimal AUC (0.9421) in the present study was achieved. Next, mRMR was applied again for the set of 290 features, which is termed as “2-mRMR,” to resolve the redundancy that exists in the selected features of different data types. However, as shown in Table 3, the number of SM remained at 110 among the new 220 features, and the AUC value was only marginally improved. These results showed that there is a large internal redundancy within one type of data, while the redundancy between different types of data is small. It further indicated that the importance of somatic mutations to the prognosis is relatively stable. Finally, we retained the integrated 290 features originally selected by mRMR and used them for further classification, considering the stable high performance and simpler simple computational complexity of mRMR. We observed that mRMR outperformed k-best methods, and integrating somatic mutations achieved the most accurate prognosis.

**TABLE 3:** Comparison of mRMR and 2-mRMR on survival prediction power and feature numbers.

	AUC(ECMPS)	Number of features					
		
		ECMPS	Exp	CNV	Methy	Protein	SM
mRMR	0.9421 ± 0.0281	290	60	50	50	20	110
2-mRMR	0.9439 ± 0.0264	220	49	22	29	10	110

#### Comparison of MKL With Traditional Classification Methods

The proposed method achieves a stronger performance by integrating somatic mutations compared with those methods incorporating single data types and integrated datasets without somatic mutations. The MKL classification method was compared with two widely used classifiers, SVM and RF, to further verify its ability to combine different types of data. Experiments were conducted in two integrated datasets: ECMP and ECMPS, which were selected by mRMR. The AUC value (mean value and standard error) was used to assess the performance of different methods and the results are provided in Table 4. Table 4 shows that a more optimal performance was obtained from MKL for both integrated datasets compared to other classifiers, and this finding indicated the superiority of MKL in data integration.

**TABLE 4:** Comparative results of the proposed MKL method and existing traditional classifiers using AUC values under two mRMR selected integrated data models.

	ECMP	ECMPS
RF	0.7135 ± 0.054	0.7916 ± 0.027
SVM	0.8325 ± 0.037	0.9086 ± 0.058
MKL	0.8578 ± 0.049	0.9421 ± 0.028

In addition, the performances of all the classifiers were improved when employing ECMPS compared with ECMP, which further suggested that somatic mutations can provide adequate supplementary information for survival prediction of breast cancer. Finally, our method achieves the most optimal performance due to its ability to integrate multiple molecular data types, including somatic mutations, and MKL was quite efficient in integrating the data from distinct sources in breast cancer survival prediction.

### Analysis of the Most Desirable Features From Somatic Mutation and Gene Expression Data

The top 10 features ranked by mRMR for each molecular data type were further analyzed by conducting a simple analysis on their association with breast cancer. Only features from somatic mutations and gene expression datasets were explored to further assess the effectiveness of our method. The results of this analysis showed that it was previously reported that some of the genes are associated with breast cancer survival. These genes and their references are listed in Table 5. It has previously been reported in the literature that seven of the top 10 ranked gene names from the somatic mutation features play critical roles in breast cancer prognosis. For example, the HCN4 gene is highly correlated with lower survival rates of breast cancer (Phan et al., 2017), and the gene PRB2 is significantly related to prognosis as an independent prognostic marker (Lv et al., 2019). On the other hand, five of the top 10 genes selected from gene expression datasets have also been found to be associated with breast cancer. For instance, the expression of IRF2 has been found to be related to breast cancer (Connett et al., 2005), and it has been reported that HMGB2 directly and significantly promotes breast cancer progression (Fu et al., 2018). Thus, the top ranked features were shown to be important for breast cancer prognosis.

**TABLE 5:** Genes previously associated with breast cancer.

Genes	Reports	References
HCN4	HCN4 was highly correlated with lower survival rates of breast cancer.	Phan et al., 2017
RGPD3	30 most enriched new HOXB7 binding sites on breast cancer cell chromatin for which an annotated nearest gene exists: RGPD3, PIK3R1, etc.	Heinonen et al., 2015
EFCAB13	Variants that induce premature stop codons were identified in the DENND2D, EFCAB13, and TICRR genes.	Määttä et al., 2016
NFATC1	NFATC1 overexpression results in oncogenic BMI1transcriptional upregulation. Co-expression of FUNDC1 and BMI1 in BC patients predicted worse prognosis.	Wu et al., 2019
VAC14	VAC14 selectively prevents rapid degradation of Sac3.	Ikonomov et al., 2013
PRB2	A novel six-gene (TMEM252, PRB2, SMCO1, IVL, SMR3B, and COL9A3) signature was significantly associated with prognosis as an independent prognostic signature.	Lv et al., 2019
HIPK1	The deletion of the miR-200c/141 cluster resulted in increased tumor metastasis and inhibited tumor growth by directly upregulating the target gene HIPK1.	Liu et al., 2018
IRF2	Interferon regulatory factor 1 (IRF-1) and IRF-2 expression in breast cancer tissue microarrays.	Connett et al., 2005
HMGB2	Promotion of breast cancer progression by HMGB2.	Fu et al., 2018
FRMPD1	Rat Mcs5a is associated with breast cancer risk. Mcs5a1 is located within the ubiquitin ligase Fbxo10, whereas Mcs5a2 includes the 5′ portion of FRMPD1.	Samuelson et al., 2007
RPS27	The best ranked cancer immunotherapy proteins related to BC were RPS27, SUPT4H1, and CLPSL2.	López-Cortés et al., 2020
PTPRR	PTPRR and myocyte enhancer factor 2C (MEF2C) genes were upregulated in the classical MAPK and p38 MAPK pathways.	Motaghed et al., 2014

### Validation

Optimization techniques have been previously applied (Zhang et al., 2016; Zhang A. et al., 2019) to select the most optimal feature subsets in a wrapper feature selection framework. Therefore, experiments were performed on an independent validation dataset to further evaluate our proposed method. Our model was initially trained and tested on a learning dataset containing 390 breast cancer patients, and then, to predict patient survival, it was applied to a 98-patient validation dataset that was not involved in training or testing. The survival of most of the 98 breast cancer patients was correctly classified, and the accuracy of the proposed method on the validation dataset was 0.9808.

## Discussion

We integrated somatic mutations and previously used data types, including Exp, CNV, Methy, and protein, using MKL to predict breast cancer patient survival. Applying mRMR-selected features and MKL classification, we found that the integration of somatic mutations enriched the diversity of features and was conducive to the improvement of the prediction model. In all, integrating promising data sources such as somatic mutations and harnessing the powerful feature selection method mRMR and the effective data fusion method MKL can increase the prediction accuracy of breast cancer patient survival.

Although our method is effective and can accurately predict the survival of breast cancer patients, some limitations remain in the prognosis of breast cancer. For instance, there may be more effective methods that can be used to construct kernels for an improved multi-kernel learning method in the future that will further improve the performance in multi-omics data fusing. In addition, our available sample size was limited by the intersection of multiple types of molecular data samples. Thus, the performance of our method could be promoted when a larger population of samples becomes available in the future. Furthermore, somatic mutations are highly heterogeneous among patients, and therefore, further understanding of the mechanism of somatic mutation in cancer may lead to a more accurate prognostic model for breast cancer.

## Data Availability Statement

The original contributions presented in the study are included in the article/Supplementary Material. Further inquiries can be directed to the corresponding author/s.

## Author Contributions

ZH and JZ participated in the design of algorithms and experiments and participated in the design of the whole framework of prediction of breast cancer survival. JZ directed the whole work. YZ participated in the analysis of the performance of the proposed method. JZ and XY conceived of the study and helped edit the manuscript. All authors read the final manuscript and approved the submission.

## Conflict of Interest

The authors declare that the research was conducted in the absence of any commercial or financial relationships that could be construed as a potential conflict of interest.

## Footnotes

1 https://xenabrowser.net/datapages/
