# Supplementary material for: Integrating Somatic Mutations for Breast Cancer Survival Prediction Using Machine Learning Methods
Source: Front Genet. 2021 Jan 18;11:632901. doi: 10.3389/fgene.2020.632901 (PMC7848170; doi:10.3389/fgene.2020.632901)
Supplement: Supplementary file 1 [file Table_1.DOCX]

**Supplement File S1.** Classification results based on mRMR and MKL using five data types and parameter determination respectively.

In our study, we used mRMR to select features with the largest relevance to the survival and lowest redundancy among themselves for each of five types of molecular data. Then the most informative features were combined as integrated features for MKL classification model. The most optimal number of finally selected simultaneously non-redundant features$k$for each molecular data were determined by AUC value of prediction results. We set $k$= [10, 20,…,300] in our experiment and chose the best optimal feature number$k$corresponding to the maximum mean value of the AUC as the final parameter for a model of further integration. The classification results of five data types under different feature numbers are shown in Figure S1. In detail, for gene expression (Exp), the optimal number of features in our model is 60, which achieves the largest mean value of AUC 0.8459 under 10-fold cross-validation. For copy number variation (CNV), methylation (Methy), protein and somatic mutation (SM) data, our model achieved the most optimal AUC values of 0.6920, 0.7465, 0.7009, and 0.8805 with the feature numbers 50, 50, 20, and 110, respectively. It should be noted that because there are only 170 proteins in our data set, the maximum number of proteins in Figure S1 is 170 instead of 300.

Finally, we chose $k$= [60, 50, 50, 20, 110] as the optimal parameters for the five types of molecular data (Exp, CNV, Methy, Protein and SM) for further integration analysis. Moreover, the most optimal classification result was obtained for somatic mutation (SM), with 110 gene mutations among the single type of data.


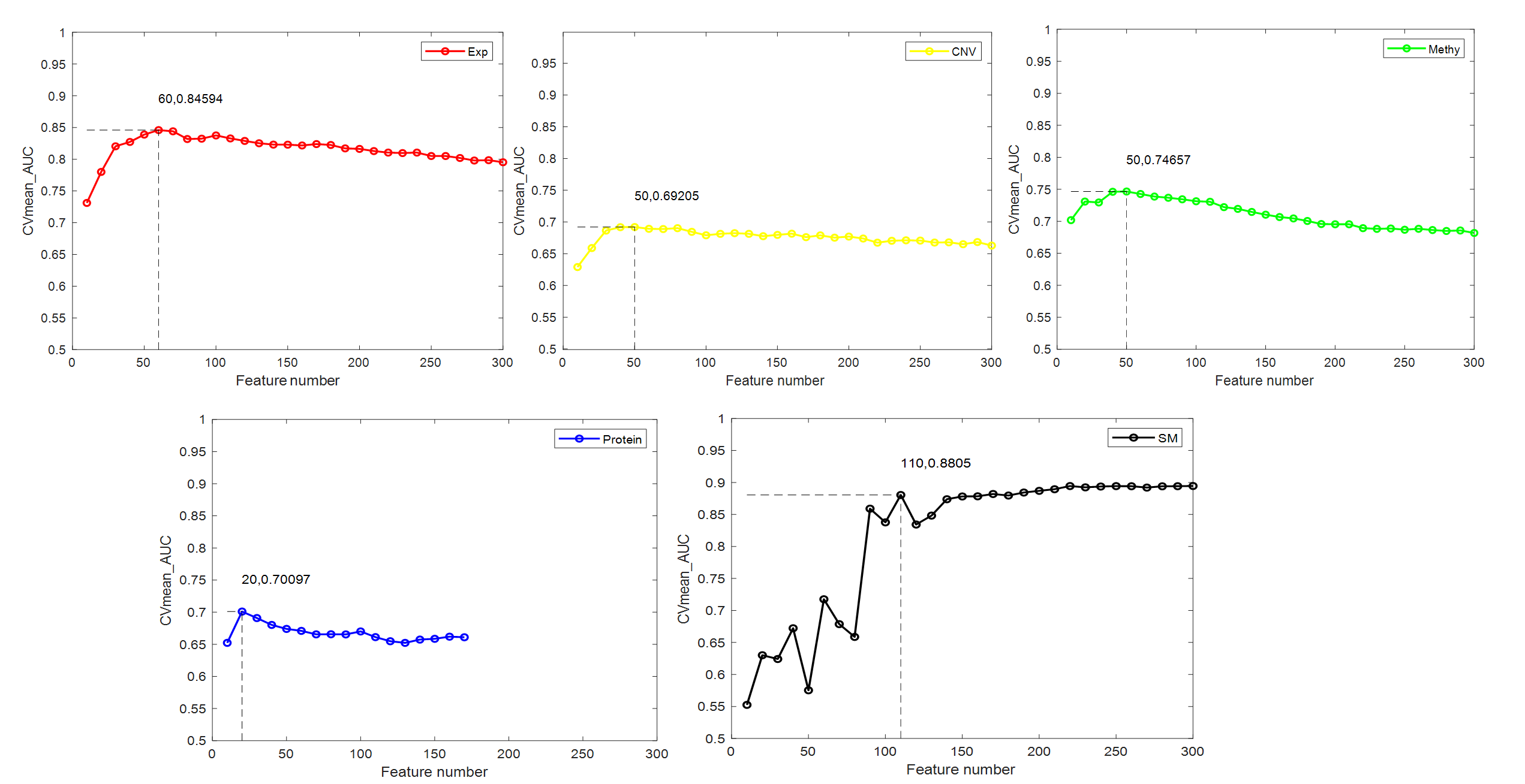


**Figure S1.** The mean value of the AUC for 10-fold cross-validation (CVmean_AUC) under the feature numbers ranging from 10 to 300 for the model based on five types of data: gene expression (Exp), copy number variation (CNV), methylation (Methy), and protein (Protein) and somatic mutation (SM) data, respectively.
